# Microfluidic Neurons, a New Way in Neuromorphic Engineering?

**DOI:** 10.3390/mi7080146

**Published:** 2016-08-22

**Authors:** Timothée Levi, Teruo Fujii

**Affiliations:** LIMMS (Laboratory for Integrated Micro Mechatronic Systems)/CNRS-IIS (Institute of Industrial Science), The University of Tokyo, 4-6-1 Komaba, Meguroku, 153-8505 Tokyo, Japan; tfujii@iis.u-tokyo.ac.jp

**Keywords:** biomimetic artificial neuron, microfluidic, action potential

## Abstract

This article describes a new way to explore neuromorphic engineering, the biomimetic artificial neuron using microfluidic techniques. This new device could replace silicon neurons and solve the issues of biocompatibility and power consumption. The biological neuron transmits electrical signals based on ion flow through their plasma membrane. Action potentials are propagated along axons and represent the fundamental electrical signals by which information are transmitted from one place to another in the nervous system. Based on this physiological behavior, we propose a microfluidic structure composed of chambers representing the intra and extracellular environments, connected by channels actuated by Quake valves. These channels are equipped with selective ion permeable membranes to mimic the exchange of chemical species found in the biological neuron. A thick polydimethylsiloxane (PDMS) membrane is used to create the Quake valve membrane. Integrated electrodes are used to measure the potential difference between the intracellular and extracellular environments: the membrane potential.

## 1. Introduction

Millions of people worldwide are affected by neurological disorders which disrupt connections between brain and body, causing paralysis or affecting cognitive capabilities. The number is likely to increase over the next few years and current assistive technology is still limited. In recent decades, extensive research has been devoted to brain-machine interfaces (BMIs), and neuroprostheses in general [1,2,3], working towards effective treatment for these disabilities. The development of these devices has had and, hopefully, will continue to have a profound social impact on these patients' quality of life. These prostheses are designed on the basis of our knowledge of interactions with neuronal cell assemblies, taking into account the intrinsic spontaneous activity of neuronal networks and understanding how to stimulate them into a desired state or produce a specific behavior. The long-term goal of replacing damaged neural networks with artificial devices also requires the development of neural network models matching the recorded electrophysiological patterns and capable of producing the correct stimulation patterns to restore the desired function. The hardware setup used to interface the biological component is a biomimetic neural network system implementing biologically realistic neural network models, ranging from the electrophysiological properties of a single neuron to large-scale neural networks. This research field is named “neuromorphic engineering”.

**Figure 1:**
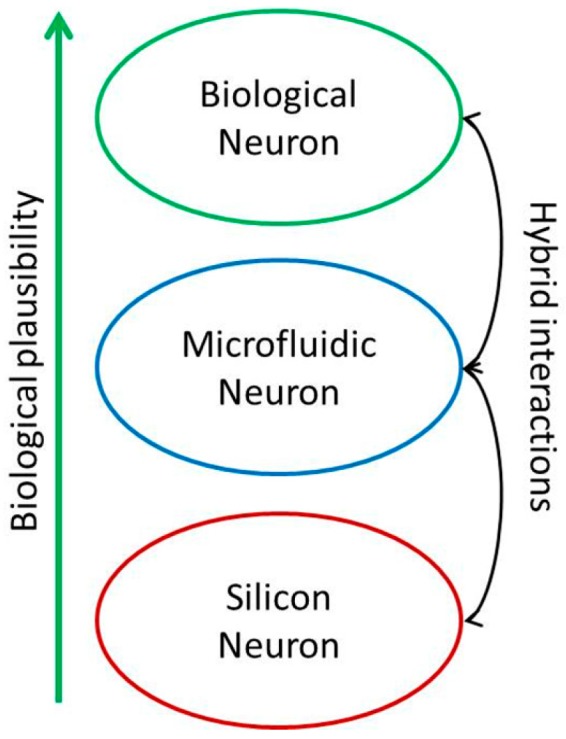
Microfluidic neuron position in the biocompatibility neuron modelling for hybrid experiments.

The neuromorphic engineering is a new emerging interdisciplinary field merging biology, physics, mathematics, computer sciences, and engineering approaches to design biomimetic artificial neural systems. These artificial neural networks emulate the electrical activity of biological neural networks and their goal is to mimic and/or replace the organic ones. Most of these systems are silicon-based [4,5]. The main goals of those systems are the design of tools for biomedical applications, like neuroprostheses [6], and the understanding of the human nervous system [7,8]. The use of silicon neurons brings some bio-compatibility issues (rejection, power consumption). To circumvent those issues, a new way of neuromorphic engineering should be explored: the artificial neural systems based on microfluidic techniques (Figure 1). The aim is to design a neuromimetic architecture of one neuron based on the use of microfluidic techniques [9] and microsensor integration. In Figure 1, the microfluidic neuron is between the silicon neuron and the biological one. It could offer a good trade-off with more biological plausibility than the silicon neuron, and to make hybrid experiments in an easier manner (bi-directional communication between a living neuron and an artificial one). To our knowledge, this new methodology does not exist yet in the state of the art.

In this article, we will first describe the novelty and the state of the art about biomimetic artificial neurons, then discuss microfluidic neuron modelling and its design; finally, we will present the first results and perspectives of this work.

## 2. Novelty and Comparison with the State of the Art in Silicon Neuron

### 2.1. Silicon Neuron for Hybrid Neural Network

In neuromorphic engineering, the most commonly used systems are silicon neurons [10]. Silicon neurons are hybrid analog/digital very large scale integration (VLSI) circuits that emulate the electrophysiological behavior of real neurons and their conductances. Silicon neuron circuits represent one of the main building blocks for implementing neuromorphic systems. The term “neuromorphic” was coined by Carver Mead [11] in the late 1980s. It refers to artificial neural systems whose architecture and design principles are based on those of biological nervous systems. The term “neuromorphic” also describes the set of analog VLSI circuits that operate using the same physics of computation used by the nervous system. It implies that the artificial neuron circuit exploits the physics of the silicon medium to directly reproduce the bio-physics of nervous cells. 

**Figure 2:**
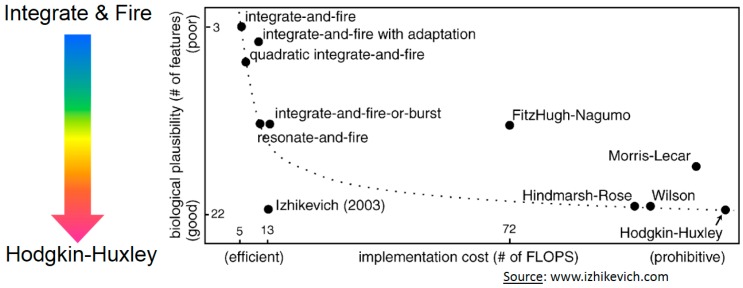
Neuron models in the literature classified following the biological plausibility and the implementation cost. The figure was extracted from www.izhikevich.com. An electronic version of the figure and reproduction permissions are freely available at www.izhikevich.com.

In designing an artificial neuron, the first step is the choice of a biologically-realistic model (Figure 2). Indeed, a mathematical model based differential equations is capable of reproducing a behavior quite similar to that of a biological cell. The choice of model is based on two criteria: the family of neurons able to be reproduced and the number of equations. Models could mimic from complex biophysical models that emulate ion channel dynamics (Hodgkin-Huxley [12]) and detailed dendritic or axonal morphologies to basic integrate-and-fire [13] circuits. 

Depending on the application domain of interest, artificial neuron circuits can be more or less complex, with large arrays of neurons all integrated on the same chip [14,15,16], or single neurons implemented on a single chip [17], or with some elements of the neuron distributed across multiple chips [18].

However, few of them [19,20,21,22,23] allow hybrid experiments with living neuron cells. The specifications of such systems, are: working in real-time with a biological time-scale, using complex neuron models that mimic the spike timing, and/or the morphology of action potential.

**Figure 3:**
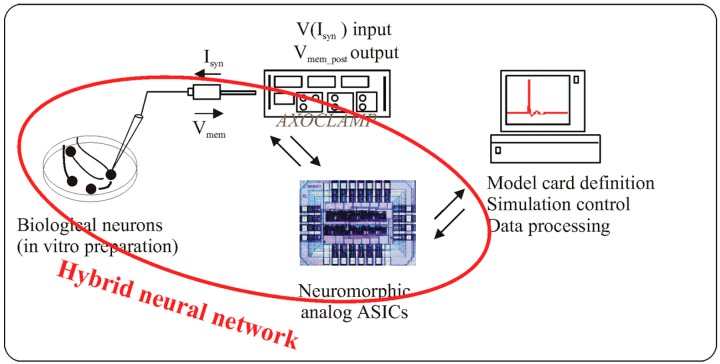
A hybrid experiment between a silicon neuron using Hodgkin-Huxley formalism and “in vitro” neurons with intracellular recording [5]. V(I_syn_) is the voltage associated to synaptic current, V_mem_post_ is the membrane voltage of the post-synaptic neuron.

Figure 3 describes one hybrid experiments and how the silicon neuron can interact with living neuronal cells. A neuron model (Hodgkin-Huxley formalism) is implemented into the ASIC (Application-Specific Integrated Circuit) which controlled the synaptic current applied to “in vitro” neuron culture. There are also some closed-loop experiments where the neural activity is recorded and controlled the artificial neural network [24].

These silicon neurons reproduce the time-spiking of the neural network but their power consumption is high. Another drawback is the difficulty of operating hybrid experiments as it needs different electrical modules like stimulation, recording, detection, amplification, and filtering modules. Several neurotechnologies for interfacing with “in vitro” neurons are already described in the literature but these system are for stimulation and/or recording of spiking activities [25,26,27,28], or for drug screening and neuroscience research [29,30,31]. They are not designed to mimic the living part and to replace it in the case of hybrid experiments. 

### 2.2. Microfluidic Neuron for Hybrid Neural Network

**Figure 4:**
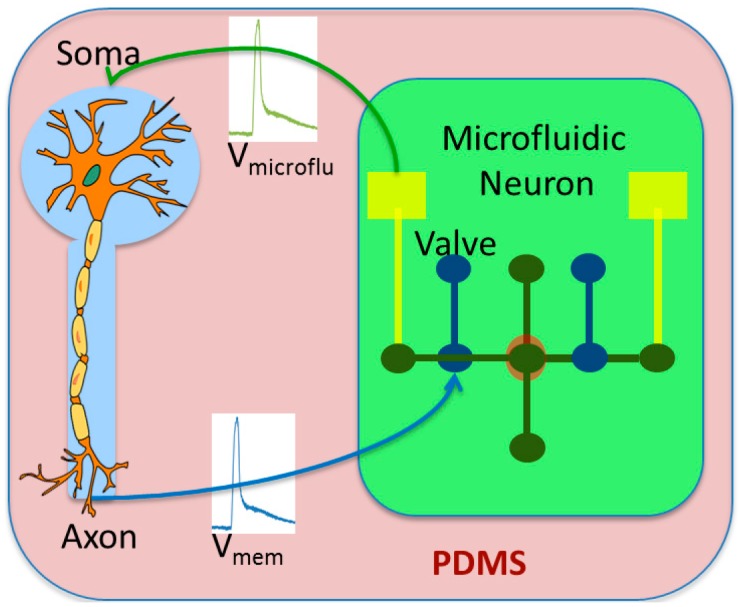
A single chip representation of a hybrid experiment using microfluidic neuron. Neuron stimulation is made from a microfluidic neuron electrode. The control of the microfluidic neuron valve is done by the electrical activity of “in vitro” neurons. V_mem_ is the action potential of the biological neuron, V_microflu_ is the action potential of the microfluidic neuron.

The main advantage of the microfluidic neuron is the biocompatibility. We can design in one single chip, the microfluidic neuron and the living neuron (Figure 4). Polydimethylsiloxane (PDMS) is made of biocompatible material and aqueous KCl and NaCl solutions are used for creating the action potential like in living neurons.

The interactions between them use the voltage membrane for stimulating each neuron. As the microfluidic neuron is using the same ionic channel than the living one, the feasibility of hybrid experiments increases.

## 3. Design of the Microfluidic Neuron

### 3.1. Neuron Modelling

**Figure 5:**
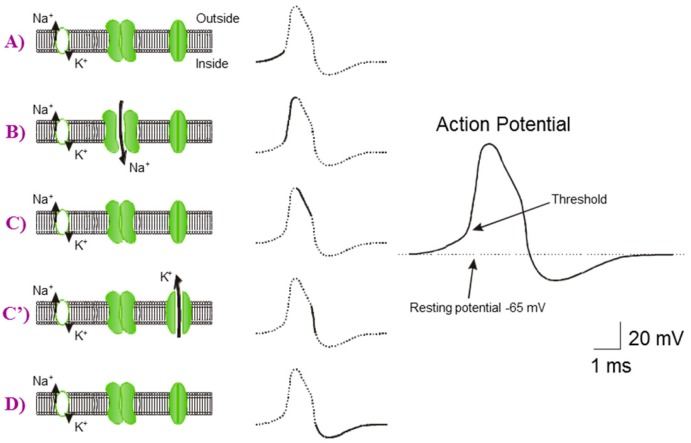
Description of the creation of action potential following the different ion interactions. (**A**) steady state of membrane voltage; (**B**) the opening of Na^+^ ionic pump depolarizes the voltage membrane; (**C**) closing of Na^+^ ionic pump; (**C’**) the opening of K^+^ ionic pump repolarizes the voltage membrane; (**D**) come back to the resting potential thanks to leakage current.

The biological neuron transmits electrical signals based on ion flow through their plasma membrane. The interior of the neurons has a negative potential, called resting potential. The action potential temporarily abolishes the resting potential negative and positive, creating the transmembrane potential. Action potentials are propagated along axons and represent the fundamental electrical signals by which information is transmitted from one place to another in the nervous system. Ion channels have an activity dependent on the potential difference between the intracellular and extracellular environments. The properties of these channels give rise to an electrical phenomenon propagating along the axon: the action potential (Figure 5).

As seen previously, there are different neuron models depending of the bioplausibility and the computation cost. The most neuromimetic model, and also the most complex, is the Hodgkin-Huxley formalism [12]. In this model, an electrical circuit has the same electrical behavior of the membrane. The different branches that make up this circuit are the membrane capacitance between extra-and intracellular environments, current generators for sodium (Na) and potassium (K) that have voltage-dependent conductances and, finally, a constant leakage channel conductance. This formalism is widely used in neuroscience as it is generalizable to any type of nerve cell. In silicon neurons, this model is hard to implement as the computation cost is prohibitive. However using microfluidic techniques, the implementation is easier than for silicon ones. We use this formalism for our microfluidic device.

### 3.2. Microfluidic Neuron

The main goals of this artificial neuron are to simplify the interactions with biological neurons, to reduce the bio-compatibility issues keeping the same ionic currents (K^+^, etc.) of biological neurons and to use biocompatible materials, like PDMS.

**Figure 6:**
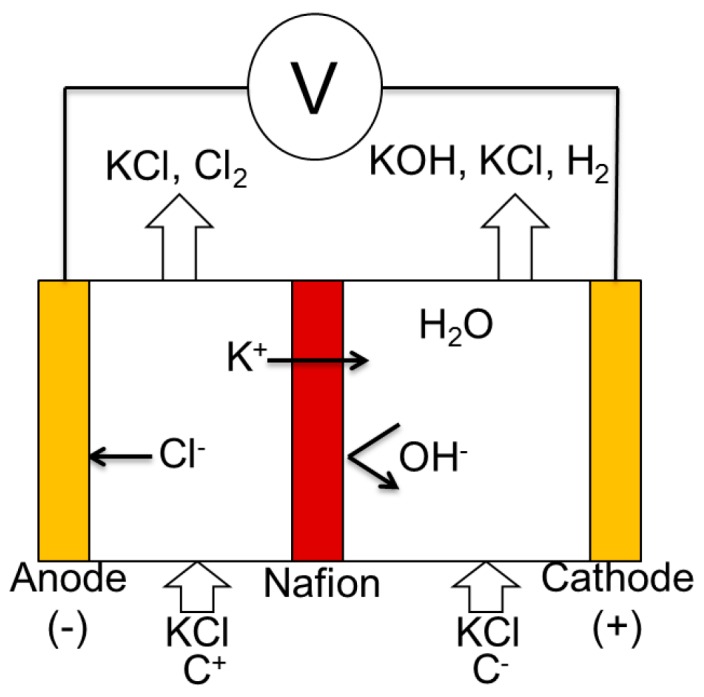
Ionic exchanges in the microfluidic neuron. C^+^ corresponds to high concentration, C^−^ to low concentration. The electrical potential form the anode and cathode mimics the action potential of one neuron. The Nafion membrane allows the flow of K^+^ but reject the negative ion, like OH^−^.

Based on the physiological behavior of the biological neuron (Figure 5) and Hodgkin-Huxley formalism [12], we propose a microfluidic structure composed of chambers representing the intra and extracellular environments, connected by channels actuated by Quake [32] valves. These channels are equipped with selective permeable membranes (Nafion) to mimic the exchange of species found in the biological neuron. In our case (Figure 6), the K^+^ is the only cation that can go through the membrane, which generates a potential when the electrons go the opposite way through the gold electrodes. The intra and extracellular chambers are controlled by pressure to control the exchange due to convection. A network of integrated gold microelectrodes is used to measure the potential difference between the intracellular and extracellular environments: the membrane potential.

A fixed potential using different ionic concentrations is generated (Nernst equations) thanks to a selective membrane. A controlled valve is also used to modify this fixed voltage potential which allows the generation of the artificial action potential.

In our experiment (Figure 6), two chambers with different KCl concentrations are separated by a selective membrane: the Nafion membrane [33]. C^+^ corresponds to high concentration, C^−^ to low concentration. The voltage potential is generated as only positive ions (K^+^ in our experiment) can go through the Nafion membrane.

**Figure 7:**
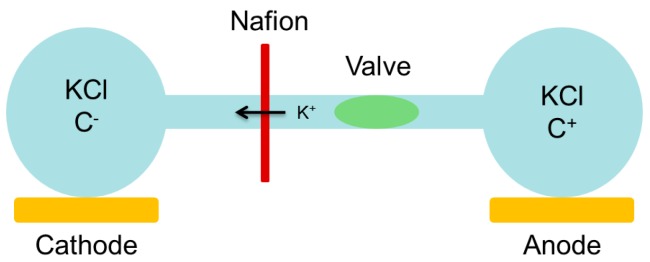
Role of the valve for the voltage potential. The open valve keeps constant voltage potential from the two chambers. The closed valve controls the amplitude of the voltage potential depending on the air-pressure used.

We generated a voltage potential between the two chambers. To modify this potential, a controlled valve is used to close the channel (Figure 7). If the valve restricts 100% of the interchamber flow, there is no more conduction and the voltage potential is null. However, as our air-pressure controlled valve cannot be fully closed, the voltage potential can be controlled by the air pressure value.

A concentration polarization is created thanks to the valves due to nanochannel modelling [34]. This phenomena seems to appear in our case and explain the variation of potential depending of the variable leakage of the channel.

### 3.3. Design Steps

To design this system, several steps are needed.

**Figure 8:**
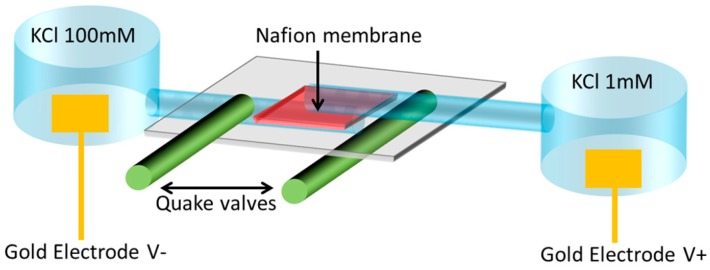
Microfluidic neuron device.

In Figure 8, the left chamber represents the intracellular environment (high concentration of K^+^) and the right chamber, the extracellular (low concentration of K^+^). Electrodes placed inside the chambers monitor the potential difference between the two fields: the membrane potential.

**Figure 9:**
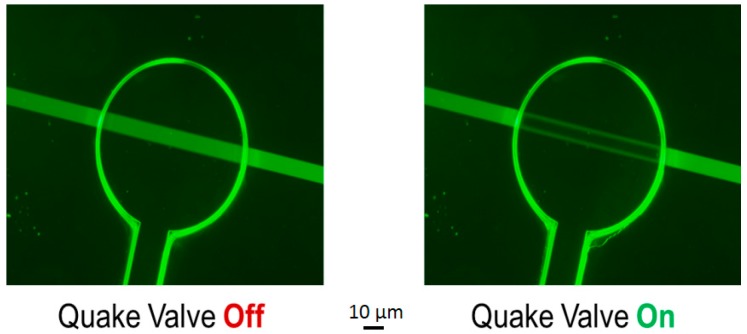
Quake valve off and on. Fluidic channel is 10 µm × 10 µm in size. The Quake valve is 20 µm × 50 µm in size. The end of Quake valve is a 1 mm diameter circle.

Exchanges between the extracellular and intracellular chambers are done through microchannels equipped with a selective membrane (Nafion) and controllable by Quake valves, as described in Figure 9. These valves are electronically controlled to reproduce the Hodgkin-Huxley formalism. When the Quake valves are activated, the channel is closed and the membrane potential is equal to 0 V.

The Quake valves are designed using a PDMS membrane of 15 µm. The air pressure channels are 20 µm × 50 µm. The liquid channels are 10 µm × 10 µm.

The Quake valves are closed using air pressure. Depending of the value of the pressure, the closing time constant can be adjusted. When the air-pressure is stopped, the valve is opened with a time constant depending of the diffusion time of the ionic solutions for recovering the ionic flow. As KCl is used, the time diffusion for K^+^ and Cl^−^ are nearly the same: K^+^ 64.7 cm/s, Cl^−^ 65.4 cm/s.

**Figure 10:**
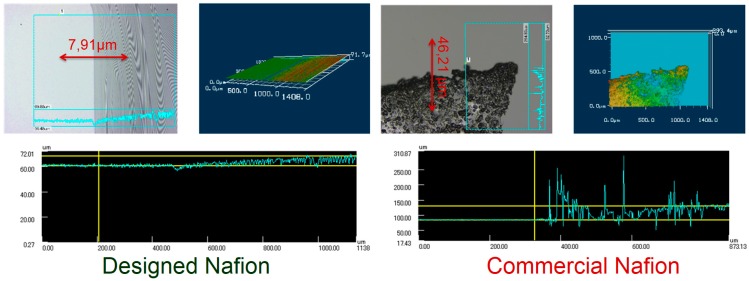
Width of Nafion membranes computed by laser confocal microscopy. In the left part, the Nafion membrane designed from Nafion solution. In the right part is a 40 µm width dry commercial Nafion membrane. The homogeneity of the left part is quite better than the right part. The commercial Nafion is also less transparent than the other one. For designed Nafion and commercial Nafion, the 3 subfigures represents the width and the surface the membrane. The top left is the computation by laser confocal microscopy (bright part is glass surface, dark part is membrane surface). The top right is the topography of the membrane showing the heterogeneity of the membrane. The bottom subfigure represents the width computation of the membrane in µm.

The selective Nafion membrane is 8 µm wide, located between the two PDMS layers of the channels. The Nafion membrane is designed with Nafion perfluorinated resin solution 5 wt % using 500 rpm spin-coating for 30 s and a 95 °C baking for 10 min. Figure 10 compares the difference between commercial Nafion membranes (dry one) where the minimum width you can buy is 40 µm. The results show that designing the Nafion membrane from Nafion solution significantly improves the quality of the membrane in terms of homogeneity, width, and transparency.

**Figure 11:**
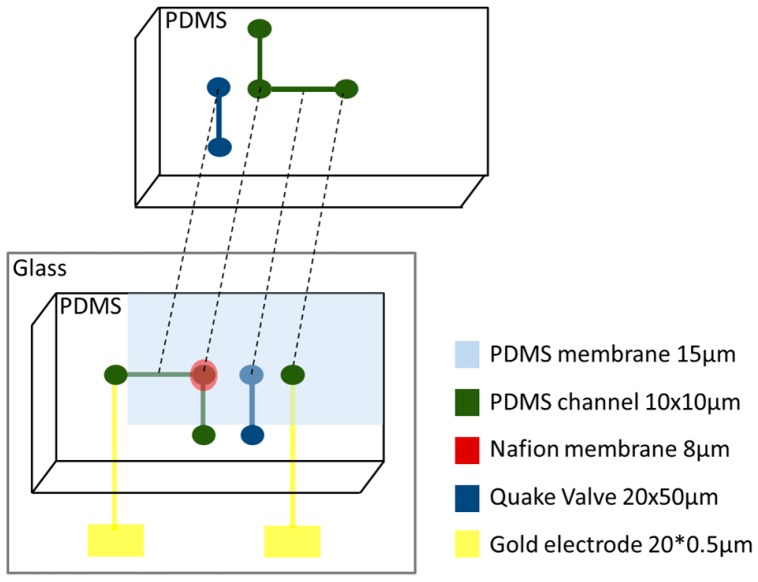
Different layers of the microfluidic neuron.

Figure 11 shows the whole microfluidic neuron device with the different layers. The size of the different channels and layers are described. The PDMS membrane is used for the Quake valve design. In this PDMS membrane we create one hole with the same size of the Nafion membrane. The interface between the two layers of channels in the center point is separated by the Nafion membrane.

**Figure 12:**
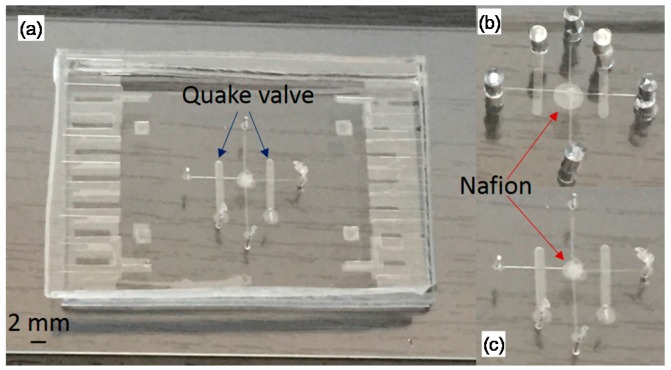
3 microfluidic neuron devices. For the device (**a**), the scale is 2 mm. For these three devices, the Nafion membrane is 10 µm width. For (**a**) and (**c**), holes are 0.5 mm in diameter. For (**b**), holes are 2 mm in diameter. The fluidic channel for these devices are 100 µm × 10 µm. The Quake valve are 500 µm × 50 µm. The PDMS membrane is 10 µm in width.

Figure 12 is composed of three pictures of microfluidic neuron devices. The diameter of the holes is equal to 0.5 mm. The holes are used for filling the four compartments (two for intracellular, two for extracellular regions) with ionic solutions. Two holes of 0.5 mm diameter are used for the air pressure inputs to open/close of the Quake valves. In Figure 12b, the holes are 2 mm in diameter in order to investigate the volume influence in the compartment. Our results did not reveal any volume influence. For these three devices, a large Quake valve was selected (500 µm) in order to investigate the size influence and also the diffusion time of the ionic solutions. Our experiments shows that the larger the Quake valve, the slower the time constant of the repolarizing phase.

## 4. Results

**Figure 13:**
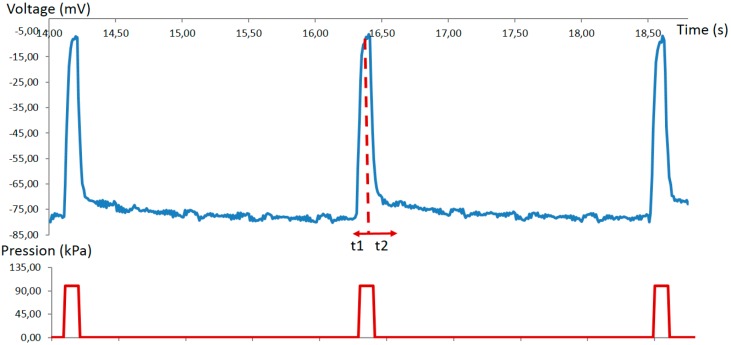
In blue, action potentials of the microfluidic neuron; in red, air pressure pulses. The action potential are created by the Quake valve using air pressure: 100 ms duration and 100 kPa pressure. Pulses are created at 14.1, 16.3 and 18.4 s. Action potential has an average amplitude of 75 mV, similar to biological neuron amplitude.

We have generated an action potential with our microfluidic neuron device (Figure 13). The three action potentials are created by air pulses of 100 ms duration and 100 kPa pressure which, respectively, close the Quake valve at 14.1, 16.3 and 18.4 s. The repolarizing phase has slower velocity than the rising phase as we observe in the biological signals.

The main parameter for controlling the shape of the action potential is the difference of ion concentration between the two chambers. In the human body, the concentration of potassium (K^+^) is 140 mM in intracellular, and 5 mM in extracellular, environments. The concentration of sodium (Na^+^) is from 5 to 15 mM in intracellular, and 145 mM in extracellular, environments. We have performed different experiments with concentrations equivalent to human ones. In a second experiment, decreased concentrations with a similar scale factor (intra and extracellular chambers) were performed. The ratio between the intra/extracellular concentrations is the most important factor, as proved by our experiments.

To conclude, we obtain an electrical behavior similar to the biological neuron. For instance, biological neuron behaviors are shown in this Nature Nanotechnology paper (Figure 3) [35]. The amplitude, waveform, and timing are similar as our experiments.

**Figure 14:**
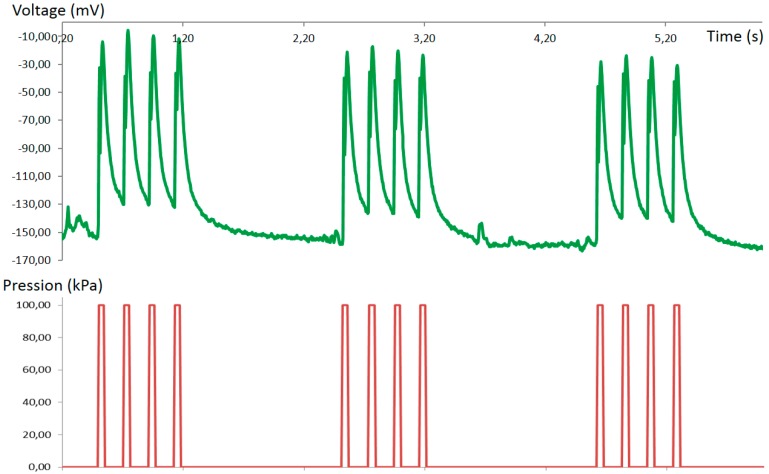
In green, burst-like behavior of the microfluidic neuron; in red, air pressure pulses. The action potentials are created by the Quake valve using air pressure: 50 ms duration and 100 kPa pressure. Shown are sequences of four pulses started at 0.50, 2.50 and 4.60 s.

Our microfluidic neuron can also generate some burst-like behavior (Figure 14). In order to get this kind of response, the opening/closure of the Quake valve has to be sequentially programmed by an air pressure controller. In our experiment, the burst frequency is lower than in biological neurons (by a factor of five). This is due to the hardware performance of our air-pressure controller.

**Figure 15:**
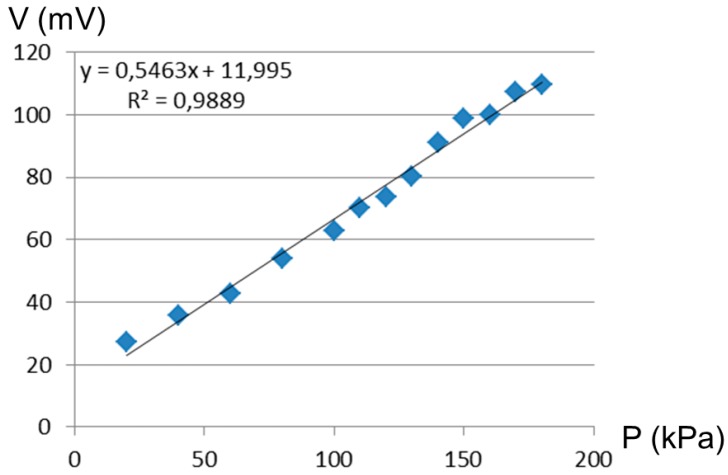
The amplitude of the action potential in function of air pressure of the Quake valves. The amplitude can be tuned from 20 to 115 mV.

The amplitude of the action potential could be determined by the air pressure value (Figure 15). The tuning of microfluidic neuron action potential amplitude is possible, whereas the behavior is difficult to obtain by a silicon one. The amplitude tuning is an important issue to mimic the biological signals. The minimum pressure is 20 kPa, as it is the limit (20 mV) for detecting the action potential from the noise. The maximum pressure was 200 kPa for our device, as more pressure cracked our PDMS membrane. This tuning is linear.

The frequency of the spiking activity can be modified by the air pressure controller. The minimum duration of this controller was 50 ms, consequently the maximum frequency of our microfluidic neuron is 20 Hz. This range is similar to biological neurons.

## 5. Discussion

### 5.1. Hybrid Experiments

**Figure 16:**
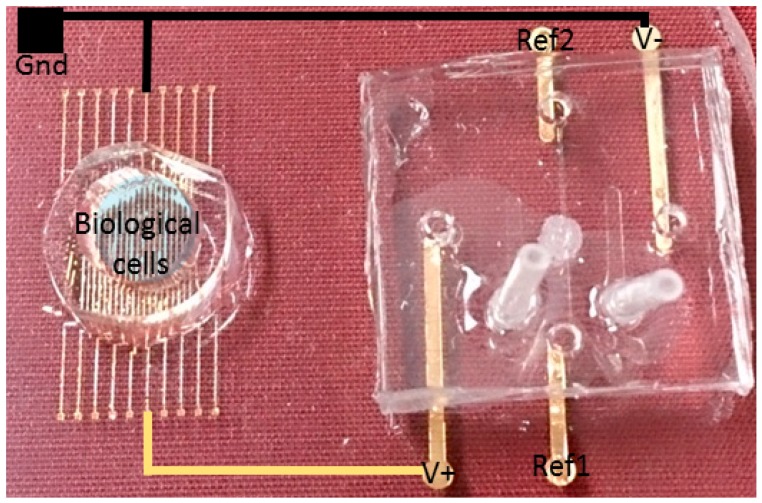
The first PDMS (polydimethylsiloxane) system for hybrid experiments. In the left part, “in vitro” neurons cultured on a gold microelectrode array (20 electrodes). In the right part, the microfluidic neuron described previously.

In a final step, hybrid experiments in the same chip were designed: neurons culture in one part of the PDMS device and artificial neurons in the other part. This PDMS device is described in Figure 16. Electrodes on glass are used for stimulation and recording of biological neurons. The microfluidic neuron is used for stimulating the biological part by the activation or inactivation of the Quake valves.

This device is ready for the open-loop (microfluidic neuron to biological neuron) experiments. For the closed-loop (bidirectional communication) experiment, an electrical board for recording and detecting the biological spiking activity is needed.

### 5.2. New Design Ideas

A key point of the design of such a microfluidic neuron is the membrane exchange between the intracellular and extracellular environments. A membrane made of Nafion provides a selectivity with respect to the charge of the ions in passing only cations (K^+^ and Na^+^ in our case). Selectivity for specific ions is not possible with this membrane. The integration of lipid bi-layers [36] would alleviate this problem, and constitute an important method to investigate.

The miniaturization of the neuron to approach the biological dimensions (of the order of tens of microns), represents an additional step to the technological constraints, particularly on the integration of the selective membrane. 

The networking of microfluidic neurons should begin before the phase of miniaturization. This will adapt to any architectural changes required to obtain neural networks with dimensions similar to those of biological neurons. 

A matrix of microfluidic neurons can be designed in the near future. The plasticity of material integration is currently under investigation in order to add synapses between the different artificial neurons.

A better miniaturization of the Quake valve can be accomplished. Bursting behavior generated by the microfluidic neuron will be more accurate and with higher frequency. The use of a piezoelectric valve could be a direction to explore.

### 5.3. Microfluidic Neuron as a Stimulator

Another potential approach will be to use the microfluidic neuron as a stimulator for biological neurons. Given that the action potential of the microfluidic neuron has the same characteristics than biological ones, and the ionic concentrations in the microfluidic neuron and in the neuron culture medium are similar, we foresee that the extracellular chamber of the microfluidic neuron and the neuron culture system can be merged. The microfluidic neuron as a stimulator is, in our opinion, one of the most promising methods to explore in neuromorphic engineering.
